# Ein Blick auf aktuelle Entwicklungen bei Blockchains und deren Auswirkungen auf den Energieverbrauch

**DOI:** 10.1007/s00287-020-01321-z

**Published:** 2020-11-06

**Authors:** Johannes Sedlmeir, Hans Ulrich Buhl, Gilbert Fridgen, Robert Keller

**Affiliations:** 1 Kernkompetenzzentrum Finanz- & Informationsmanagement, Projektgruppe Wirtschaftsinformatik des Fraunhofer FIT, Universität Bayreuth, Bayreuth, Deutschland; 2 Kernkompetenzzentrum Finanz- & Informationsmanagement, Projektgruppe Wirtschaftsinformatik des Fraunhofer FIT, Universität Augsburg, Augsburg, Deutschland; 3 SnT – Interdisciplinary Center for Security, Reliability and Trust, University of Luxembourg, Luxemburg, Luxemburg

## Abstract

Der enorme Stromverbrauch von Bitcoin hat dazu geführt, dass in Wissenschaft und Praxis oft eher undifferenziert Diskussionen über die Nachhaltigkeit von Blockchain- bzw. Distributed-Ledger-Technologie allgemein geführt werden. Allerdings ist die Blockchain-Technologie bereits heute alles andere als homogen – nicht nur hinsichtlich ihrer Anwendungen, die mittlerweile weit über Kryptowährungen hinaus in Wirtschaft und öffentlichen Sektor reichen, sondern auch bezüglich ihrer technischen Charakteristika und insbesondere ihres Stromverbrauchs. Dieser Beitrag fasst den Status quo des Stromverbrauchs verschiedener Implementierungen von Blockchain-Technologie zusammen und geht dabei besonders auf das kürzlich erfolgte Bitcoin Halving sowie sogenannte ZK-Rollups ein. Wir argumentieren, dass Bitcoin und andere Proof-of-Work-Blockchains zwar in der Tat sehr viel Strom verbrauchen, aber bereits heute alternative Blockchain-Lösungen mit deutlich geringerem Stromverbrauch verfügbar sind und weitere vielversprechende Konzepte erprobt werden, die gerade den Stromverbrauch von großen Blockchain-Netzwerken in naher Zukunft noch einmal deutlich senken könnten. Daraus schließen wir, dass die Kritik am Stromverbrauch von Bitcoin zwar legitim ist, jedoch daraus nicht eine Energieproblematik von Blockchain-Technologie generell abgeleitet werden darf. In vielen Fällen, in denen mithilfe von energieeffizienteren Blockchain-Varianten Prozesse digitalisiert oder verbessert werden können, darf sogar per Saldo durchaus mit Energieeinsparungen gerechnet werden.

## Abstract (en)

The enormous power consumption of Bitcoin has led to undifferentiated discussions in science and practice about the sustainability of blockchain and distributed ledger technology in general. However, blockchain technology is far from homogeneous—not only with regard to its applications, which now go far beyond cryptocurrencies and have reached businesses and the public sector, but also with regard to its technical characteristics and, in particular, its power consumption. This paper summarizes the status quo of the power consumption of various implementations of blockchain technology, with special emphasis on the recent ‘‘Bitcoin Halving’’ and so-called ‘‘zk-rollups’’. We argue that although Bitcoin and other proof-of-work blockchains do indeed consume a lot of power, alternative blockchain solutions with significantly lower power consumption are already available today, and new promising concepts are being tested that could further reduce in particulary the power consumption of large blockchain networks in the near future. From this we conclude that although the criticism of Bitcoin’s power consumption is legitimate, it should not be used to derive an energy problem of blockchain technology in general. In many cases in which processes can be digitized or improved with the help of more energy-efficient blockchain variants, one can even expect net energy savings.

## Einleitung

In führenden deutschen Printmedien kann man Aussagen wie „[m]ittlerweile verbraucht das Bitcoin-System in etwa so viel Strom wie die Bundesrepublik, Tendenz steigend“ (Frankfurter Allgemeine Zeitung vom 06.06.2020 [1]) begegnen. Auf der anderen Seite wurde 2018 in der Zeitschrift *Nature Climate Change* ein Artikel veröffentlicht, demzufolge bei großflächiger Adoption von Bitcoin alleine die dadurch in den kommenden 3 Dekaden verursachten Emissionen zu einer Erderwärmung um mehr als 2 °C führen könnten [2]. Der FAZ-Artikel wurde zwar auf unsere Initiative hin bereits kurze Zeit nach seiner Veröffentlichung in der Onlineversion modifiziert und dem Nature-Artikel folgte eine kontroverse wissenschaftliche Diskussion über die Sinnhaftigkeit der zugrunde liegenden Annahmen. Dennoch führen solche Publikationen in der Öffentlichkeit zu einem unzutreffenden Eindruck bezüglich der ökologischen Folgen von Bitcoin und zu einer noch problematischeren Verallgemeinerung auf Blockchains.

Im Kern ist die Aussage, dass Bitcoin und auch viele andere Kryptowährungen einen enormen Stromverbrauch verursachen, richtig und wichtig und wurde in zahlreichen Publikationen, unter anderem in den Zeitschriften *Joule* [3–5] und *Nature Sustainability* [6], im Detail diskutiert und begründet. Häufig aber bleiben gerade die plakativen Aussagen präsent, werden aus dem Zusammenhang gerissen, unzutreffend verallgemeinert oder für Argumentationslinien verwendet, die von einem fehlenden Verständnis der grundlegenden Zusammenhänge zwischen dem hohen Stromverbrauch einiger Kryptowährungen und ökonomischen sowie technischen Parametern zeugen. Beispielsweise steigt der Stromverbrauch von Bitcoin weder zwangsläufig stetig an noch wächst er signifikant mit der Anzahl an abgewickelten Transaktionen pro Zeiteinheit. Darüber hinaus wird Blockchain-Technologie sowohl in der öffentlichen Berichterstattung als auch teilweise in der Wissenschaft so häufig in einem Atemzug mit Bitcoin genannt und für die Erläuterung der Funktionsweise von Blockchains herangezogen, dass sich daraus gewisse Vorurteile hinsichtlich des Stromverbrauchs von Blockchain-Technologie allgemein etablieren konnten.

Tatsächlich gibt es mittlerweile zahlreiche Kryptowährungen, die auf technisch deutlich veränderten Blockchain-Varianten mit vollkommen anderen Charakteristika hinsichtlich ihres Stromverbrauchs basieren. Ähnlich verhält es sich bei einer Vielzahl von Implementierungen blockchain-basierter Plattformen für organisationsübergreifende Prozesse in Wirtschaft und öffentlichem Sektor. In Deutschland sind hier etwa Projekte von Automobilherstellern im Supply-Chain-Bereich [7] oder dem Bundesamt für Migration und Flüchtlinge [8] zu nennen. Da das Thema Nachhaltigkeit in Politik und Wirtschaft zu Recht sehr präsent ist [9], wird im Zusammenhang mit blockchain-bezogenen Projekten aus den oben beschriebenen Gründen sehr häufig die Frage nach dem Stromverbrauch und der Nachhaltigkeit von Blockchain-Technologie generell gestellt. Die Präsenz des Stromverbrauchstigmas könnte daher die Adoption von Blockchain-Technologie und damit die Nutzung ihrer Vorteile merklich behindern [10].

Entsprechend wollen wir in diesem Beitrag einen umfassenden Überblick über den Stromverbrauch von Blockchain-Technologie allgemein geben, um eine solide Diskussionsgrundlage für den allgemeinen Diskurs zu schaffen. Dazu beschreiben wir zunächst gut bekannte Abschätzungen für den Energieverbrauch von Bitcoin, erweitern diese aber durch eine detaillierte Diskussion des kürzlich erfolgten Bitcoin Halving, welches viele der grundlegenden Zusammenhänge offenbart. Beim Bitcoin Halving wird periodisch etwa alle 4 Jahre die Anzahl der pro Block neu geschaffenen und dem Miner als Belohnung dienenden Bitcoins halbiert. Dadurch wird sichergestellt, dass die Anzahl der existierenden Bitcoins beschränkt bleibt (geometrische Reihe). Ziel dieser Konstruktion ist, die Inflation zu reduzieren. Zum anderen untersuchen wir auch einen erheblich größeren Teil des wie beschrieben sehr heterogenen Spektrums von Blockchains als nur einige technisch eng mit Bitcoin verwandte Kryptowährungen. Damit erweitern wir einen von uns in der Zeitschrift *Business & Information Systems Engineering* veröffentlichten Beitrag [11] zum Energieverbrauch von Blockchains, welcher bereits einige der in diesem Artikel angesprochenen Punkte diskutiert und sich dabei stärker auf die Nachhaltigkeitsdiskussion über Kryptowährungen hinausgehender Anwendungen von Blockchain-Technologie fokussiert. Im Vergleich dazu gehen wir hier auf einige dort nur kurz andiskutierte Aspekte wesentlich detaillierter ein, insbesondere analysieren wir neben dem Bitcoin Halving quantitativ die Implikationen der Verwendung sogenannter ZK-Rollups auf den Stromverbrauch von Blockchains.

Trotz der Tatsache, dass Blockchain-Technologie weit vielfältiger eingesetzt wird als in Bitcoin und Kryptowährungen, nimmt Bitcoin auch in diesem Artikel eine zentrale Stellung ein. Dies ist dessen problematisch hohem Energiebedarf geschuldet. Andere Anwendungen von Blockchain-Technologie sind nach unserer Überzeugung wesentlich bedeutungsvoller.

## Grundlagen von Bitcoin und Blockchain-Technologie

Die Bitcoin-Blockchain wurde entwickelt, um ein dezentrales, elektronisches Währungssystem zu schaffen. (Vermögens‑)Werte zu übertragen ist – im Gegensatz zum Übertragen von Informationen – elektronisch nicht ohne Weiteres bilateral möglich, da elektronische Objekte ohne Aufwand praktisch beliebig oft kopiert werden können. Daher kann zwar die in den elektronischen Objekten enthaltene Information an sich wertvoll sein, durch die Übergabe oder das Abspeichern des elektronischen Objekts an sich wird aber kein Wert übertragen [12]. Entsprechend benötigt man zur elektronischen Übertragung von Werten innerhalb einer bestimmten Gruppe ein von allen Mitgliedern anerkanntes sogenanntes Register (Ledger), in das die Eigentumsverhältnisse eingetragen werden. Eine Änderung dieser Eigentumsverhältnisse in einem elektronischen Register kann also als elektronische Wertübertragung oder Transaktion verstanden werden.

Traditionell werden solche Register in Form von Datenbanken stets von vertrauenswürdigen Dritten, im Währungskontext etwa Banken geführt. Die 2008 in einem White Paper vorgestellte Kryptowährung Bitcoin [13], die anschließend implementiert wurde und 2009 auch in Betrieb ging, beruht dagegen auf einer dezentralen Verwaltung des entsprechenden elektronischen Registers durch redundante und synchronisierte („physisch dezentrale, logisch zentrale“) Haltung des Registers auf allen beteiligten Rechnern (Nodes). Dadurch wird über die Gültigkeit und das Ausführen von Transaktionen nun nicht mehr nur durch eine zentrale Instanz, sondern durch alle Teilnehmer am Bitcoin-Netzwerk entschieden. Die Authentifizierung, etwa zum Nachweis von Eigentum an Einheiten der Kryptowährung oder der Autorisierung von Bezahlungen durch diesen Account, geschieht dabei mithilfe einer Public-Key-Infrastruktur und entsprechender digitaler Signaturen. Zum Zweck der Mehrheitsfindung für Entscheidungen benötigt das Bitcoin-Netzwerk einen sogenannten Konsensmechanismus, nach dem die Nodes entscheiden, welche neuen Transaktionen in welcher Reihenfolge aufgenommen werden.

Im Prinzip wurden solche Replicated State Machines, die auch in Gegenwart von Systemausfällen oder byzantinischen Fehlern Sicherheit und Funktionsfähigkeit garantieren, seit 1982 intensiv erforscht [14] und mit Paxos [15] und PBFT [15] und PBFT [16] dann auch praktisch implementiert. Konsens konnte dabei wahlbasiert getreu dem Motto „ein Node, eine Stimme“ gefunden werden. Neu ist bei Bitcoin jedoch, dass nicht nur vorab definierte Nodes an dem Netzwerk und entsprechend an der Konsensfindung teilnehmen können, sondern jeder, der möchte. Dies bezeichnet man als ein offenes, nicht zugangsbeschränktes System. Darin ist der soeben beschriebene wahlbasierte Prozess nicht möglich, da ein Angreifer, der das System überstimmen möchte, nur ausreichend viele Accounts im Netzwerk registrieren müsste, was für diesen ohne nennenswerte Kosten möglich wäre (man nennt dies Sybil Attack, siehe z. B. [17]).

Ein System ohne Zugangsbeschränkung wie Bitcoin muss daher das Gewicht einer Stimme beim Abstimmen an eine knappe Ressource binden, um solche Angriffe zu verhindern. Bei Bitcoin und vielen anderen Kryptowährungen geschieht dies über den sogenannten Proof of Work (PoW), d. h., das Gewicht der Stimme wird an nachgewiesene, geleistete Rechenarbeit und damit Energie gekoppelt. Der Nachweis von Rechenarbeit besteht hierbei in dem Finden einer Zufallszahl, der sogenannten Nonce, sodass der Hashwert der Nonce – gemeinsam mit weiteren Daten – eine bestimmte Form annimmt. Im Falle von Bitcoin ist dies die Forderung, dass die Ganzzahlrepräsentierung des Hashwertes kleiner als eine bestimmte Obergrenze ist. Die Wahl dieser Obergrenze definiert dadurch eine Schwierigkeit, die sogenannte Difficulty, dieses kryptografischen Rätsels. Die Difficulty ist indirekt proportional zur Wahrscheinlichkeit, dass eine zufällig gewählte Nonce zu einem Hashwert der gewünschten Form führt. Diese Methode zum Nachweis erbrachter Rechenleistung ist bereits seit Langem bekannt und wurde etwa in Hashcash für das Verhindern von Spam diskutiert [18].

Die Teilnahme am PoW-Konsensmechanismus, also das Suchen nach entsprechenden Nonces, ist demnach mit Kosten verbunden, sodass ein ökonomischer Anreiz für die Teilnahme am Mining geschaffen werden muss: Wer eine Nonce findet, die gemeinsam mit einem Bündel an Transaktionen zu einem Hashwert der erforderten Form führt, darf auch eine Belohnung in Höhe einer gewissen Zahl an für diesen Zweck neu geschaffenen Bitcoins für sich eintragen (Block Reward). Den entsprechenden Block kann man dann an die anderen Teilnehmer im Blockchain-Netzwerk kommunizieren und damit an die bestehende Kette anhängen, was bedeutet, dass die entsprechenden Transaktionen ausgeführt werden. Dabei ist zu erwähnen, dass aufgrund des Wettbewerbs beim Bitcoin-Mining mittlerweile die Teilnahme mit CPUs längst unrentabel ist, da spezialisierte Hardware, sogenannte ASICs, entwickelt wurden, die Hashes um Größenordnungen schneller und energieeffizienter berechnen können als CPUs und GPUs [3]. Der Unterschied ist bei Bitcoin so gravierend, dass sogar die 500 weltweit größten Supercomputer zusammengenommen vermutlich nur einen kleinen Teil der aktuellen, überwiegend auf ASICs Hashrate von Bitcoin erreichen können – und das auch nur unter erheblichen finanziellen Verlusten.

Um unerkannte Manipulationen im System zu verhindern, verwendet Bitcoin eine Datenstruktur, die das Erkennen und Auffinden von nachträglichen Veränderungen sehr leicht macht, nämlich Merkle Trees. Um den Aufwand bei der Konsensfindung zu reduzieren, werden zahlreiche Transaktionen, Metadaten, Nonce sowie ein Hashpointer auf den vorherigen Block in einem Block zusammengefasst. Die resultierende Append-Only-Struktur (Chain) erhält dadurch die Eigenschaft, dass die Änderung nur einer einzigen Transaktion entweder zur Inkonsistenz eines einzelnen Blocks (falsche Merkle Root) führt oder alle Hashpointer ab dem manipulierten Block ebenfalls geändert werden müssten. Durch die Forderung, dass der Hashwert jedes Blocks die oben beschriebene Form haben muss, ist das Finden solcher Blöcke und damit einer alternativen Kette von Hashpointern sehr rechenaufwendig, sodass das System sicher ist, solange ein Großteil der Hashrate von „ehrlichen“ Nodes gestellt wird (zu Angriffsszenarien siehe etwa [19]). Die Datenstruktur aus Blöcken und Hashpointern ist allgemein charakteristisch für die Blockchain-Technologie, welche wiederum ein Spezialfall von sogenannten Distributed-Ledger-Technologien ist. Üblicherweise versteht man jedoch unter Blockchain-Technologie nicht nur diese Datenstruktur, sondern auch das Vorhandensein eines Konsensmechanismus, der sowohl eine Einigung über das Hinzufügen neuer Transaktionen ermöglicht als auch sicherstellt, dass keine nachträglichen Änderungen an der Blockchain durchgeführt werden können. Hierfür können jedoch neben der in PoW genutzten Schwierigkeit, Urbilder bestimmter Hashwerte zu finden, auch andere Methoden angewendet werden. Dies sind in der Regel digitale Signaturen, die je nach Konsensmechanismus von entweder nach festen Regeln oder (pseudo-)zufällig bestimmten Netzwerkteilnehmern erstellt werden. Auf diese Varianten werden wir bei der Diskussion alternativer Konsensmechanismen noch kurz eingehen.

## Abschätzungen für den Energieverbrauch von PoW-Blockchains

Wie bereits beschrieben, besteht der Anreiz für die Teilnahme am Mining in der Bitcoin-Blockchain und allgemein bei PoW-basierten Blockchains in der Belohnung in Form von Einheiten der damit als Anreizsystem nötigen, dazugehörigen („nativen“) Kryptowährung. Durch die starken Kurssteigerungen von Kryptowährungen mit dem Höhepunkt Ende 2017 und einer Marktkapitalisierung von kurzzeitig über 300 Mrd. und seitdem stets über 50 Mrd. US-Dollar alleine von Bitcoin gibt und gab es also einen großen ökonomischen Anreiz für die Teilnahme am Mining. Um die Funktionalität (und auch die Sicherheit) des PoW-Blockchain-Netzwerks aufrechtzuerhalten, muss die Zeitspanne, in der üblicherweise ein neuer Block gefunden wird, konstant gehalten werden, d. h., die Schwierigkeit des Hashpuzzles muss entsprechend der aktuellen Hashrate angepasst werden. Dies führt zu einem entsprechend hohen Stromverbrauch von PoW-basierten Kryptowährungen.

Grundsätzlich ist die exakte Bestimmung des Stromverbrauchs in einer offenen und nicht zugangsbeschränkten PoW-Blockchain sehr schwierig, da man in der Regel weder die beim Mining eingesetzte Rechenleistung noch die entsprechende Hardware für jeden einzelnen Teilnehmer bestimmen kann. Eine untere Grenze für den Stromverbrauch von Bitcoin und allen anderen PoW-basierten Blockchains kann man jedoch leicht aus der indirekt beobachtbaren mittleren Rechenleistung, also der Hashrate, und der energieeffizientesten Mining-Hardware auf dem Markt bestimmen [3, 20]. Dabei kann man den Erwartungswert der Hashrate aus der öffentlich einsehbaren aktuellen Schwierigkeit des Hashpuzzles sowie der Anzahl der in Form von neuen Blöcken kommunizierten Lösungen abschätzen. Bei Bitcoin wird nach Konstruktion des Protokolls im Mittel alle 10 Minuten eine neue Lösung des Hashpuzzles gefunden und die Wahrscheinlichkeit, dass ein zufälliger Hashwert die Anforderungen erfüllt, lag Anfang 2020 bei etwa $1:6\times 10^{22}$. Für Bitcoin wird SHA-256 als Hashing-Algorithmus verwendet; moderne ASICs erreichen dafür Hashraten in der Größenordnung von $10^{14}$ Hashes pro Sekunde bei einer Leistung von wenigen Tausend Watt. Daraus erhält man für den Strombedarf von Bitcoin Anfang 2020 eine untere Schranke von jährlich ca. 60 TWh [11], was dem jährlichen Stromverbrauch von ca. 15 Mio. Haushalten entspricht.

Eine obere Schranke für den durch das Mining bewirkten Stromverbrauch kann man ebenfalls abschätzen, solange man annimmt, dass alle Teilnehmer am Konsensmechanismus rational handeln und durch die Teilnahme am Mining Gewinn anstreben. Dies mag nicht für alle Teilnehmer stimmen, aber die überwiegende Mehrheit der Rechenleistung wird bei Bitcoin und anderen relevanten PoW-Kryptowährungen von auf Mining spezialisierten Unternehmen oder Gruppen (Pools) gestellt [21], für die diese Annahme sinnvoll erscheint. Der Wert des ökonomischen Anreizes, also der durch Mining neu erzeugten Bitcoins, muss im Mittel mindestens so hoch sein wie für die durch das Mining verursachten Kosten, also etwa für elektrische Energie und Hardware, und damit insbesondere höher als die Stromkosten. Eine untere Schranke für Kosten der elektrischen Energie in Ländern mit nennenswerter Beteiligung am Mining wird üblicherweise mit 0,05 USD pro Kilowattstunde beziffert [22, 23] und dadurch erhält man für Anfang 2020 bei einem Bitcoin-Preis von knapp 10.000 USD hochgerechnet eine Obergrenze des Stromverbrauchs von jährlich ca. 120 TWh, was etwa 20 % des deutschen Stromverbrauchs entspricht [11].

Für andere, bekannte PoW-Blockchains, wie etwa Ethereum, Bitcoin Cash, Bitcoin SV und Litecoin (dies sind nach Bitcoin die nach Marktkapitalisierung größten PoW-basierten Kryptowährungen), gelten dieselben Abschätzungsformeln wie für Bitcoin, nur liegen hier andere Hashing-Algorithmen, spezialisierte Mining-Hardware und Parameter wie mittlere Blockzeiten und Block Rewards vor. In Summe beträgt der Stromverbrauch der genannten 4 Kryptowährungen zwischen 10 TWh und 40 TWh pro Jahr und ist damit deutlich niedriger als der von Bitcoin. Man stellt zudem fest, dass wegen im Allgemeinen ähnlicher Parameter bei verschiedenen PoW-Kryptowährungen eine sehr hohe Korrelation zwischen Marktkapitalisierung und Stromverbrauch vorhanden ist. Da die Marktkapitalisierung von Bitcoin höher ist als die kumulierte aller übrigen Kryptowährungen, kann man vermuten, dass der kumulierte Stromverbrauch aller PoW-Kryptowährungen nicht viel mehr als doppelt so hoch ist wie der von Bitcoin, und ein Best Guess liegt bei einem Faktor von etwa 1,5 [4, 11].

Eine wichtige Beobachtung für PoW-Kryptowährungen ist, dass sich deren Stromverbrauch langfristig nicht durch Steigerungen der Energieeffizienz von Hardware senken lässt: Zum einen kann man dies daran sehen, dass die Abschätzung der oberen Schranke nur von den Strompreisen und nicht von der Rechenleistung abhängt. Der Grund dafür ist, dass langfristig alle Miner auf energieeffizientere Hardware umsteigen würden, solange Mining damit profitabel ist. Entsprechend steigt wie bereits beschrieben auch die Gesamtrechenleistung des Netzwerks, bis das Gleichgewicht der Einnahmen- und Ausgabenseite wieder näherungsweise hergestellt ist.

Wegen des Anspruchs, möglichst vielen Nodes eine Teilnahme an Kryptowährungen zu ermöglichen, sowie der redundanten Ausführung aller Transaktionen müssen die technischen Anforderungen zur Teilnahme, also Netzwerkbandbreite und Speicherplatz, möglichst gering gehalten werden. Da der „langsamste“ zulässige Node die Performanz des Systems vorgibt, können Bitcoin und andere Kryptowährungssysteme nur wenige Transaktionen pro Sekunde abwickeln – aktuell benötigt der für die komplette Bitcoin-Blockchain benötigte Speicherplatz knapp 300 GB und wächst um ca. 60 GB pro Jahr, eine Vervielfachung der Transaktionen pro Zeiteinheit würde das Wachstum ebenfalls vervielfachen. Entsprechend fällt bei einfacher Division des Stromverbrauchs durch die Anzahl der Transaktionen bei PoW-basierten Kryptowährungen je Transaktion eine enorme Energiemenge an: Für Bitcoin beträgt der Stromverbrauch für eine einzelne Transaktion dann mehrere Hundert kWh und entspricht damit dem Stromverbrauch eines durchschnittlichen deutschen Haushalts von mehreren Wochen bis Monaten, was zu der beschriebenen häufigen Kritik an der Nachhaltigkeit von Bitcoin führt. Für andere PoW-basierte Kryptowährungen erhält man zwar deutlich niedrigere Werte je Transaktion, dennoch ist dies noch immer um Größenordnungen energieintensiver als beispielsweise eine herkömmliche Buchung im Bankensystem. Es ist jedoch essenziell, zu verstehen, dass die Anzahl an abgewickelten Transaktionen keine Auswirkung auf den durch das Mining bedingten Stromverbrauch des Gesamtnetzwerks hat, da in der Theorie die Blöcke beliebig vergrößert werden könnten [24]. Somit ist die Metrik „Energie pro Transaktion“ für PoW-basierte Kryptowährungen durchaus ambivalent zu betrachten. Nichtsdestotrotz kann man angesichts der Leistungsfähigkeit von Bitcoin und auch anderer aktueller PoW-Blockchains deren Stromverbrauch durchaus als unverhältnismäßig bezeichnen.

## Ausblick: Implikationen aus dem kürzlich erfolgten Bitcoin Halving

**Figure 1:**
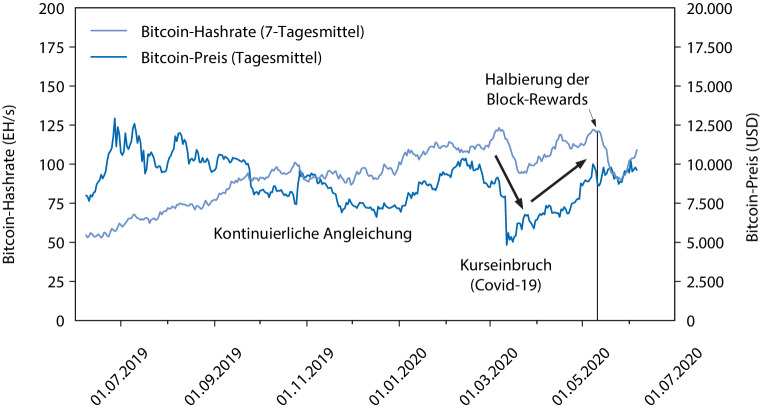

Im Folgenden werden durch eine Analyse des kürzlichen Bitcoin Halving eine detailliertere Analyse des Stromverbrauchs von Bitcoin durchgeführt sowie daraus Implikationen für die langfristige Entwicklung des Stromverbrauchs abgeleitet. Der in Abb. 1 dargestellte Vergleich der Entwicklung der Bitcoin-Preise und Hashrate der vergangenen 12 Monate legt nahe, dass die oben beschriebene obere Schranke tatsächlich eine recht gute Schätzung für den tatsächlichen Stromverbrauch darstellt: Bei relativ stabilen Bitcoin-Preisen bis März 2020 steigt die beobachtete Hashrate kontinuierlich an; offenbar wurden hier das Aufnehmen oder Ausweiten von Mining-Aktivitäten, was mit der Beschaffung von entsprechender Hardware verbunden ist, als lohnenswert angesehen. Ein Kurssturz von Bitcoin Anfang März 2020 im Rahmen einer allgemein schwachen Börsenstimmung infolge der COVID-19-Pandemie wurde jedoch von einem zwar etwas geringer ausgeprägten, aber dennoch deutlichen Abfall der Hashrate begleitet. Man könnte dies dadurch erklären, dass wegen der Reduktion des Werts von Bitcoin und damit der Höhe des Mining-Anreizes Miner mit höheren variablen Kosten, etwa aufgrund veralteter Hardware oder hoher Strompreise, hier kurzfristig aus dem Mining gedrängt wurden. Danach stieg mit dem Bitcoin-Kurs auch die Hashrate wieder auf das vorherige Niveau. Das in vielen PoW-Blockchains vorgesehene und im Falle von Bitcoin etwa alle 4 Jahre geschehende Bitcoin Halving am 11.05.2020 bewirkte dann jedoch eine permanente Halbierung der Block-Rewards und eine entsprechde Reduktion des ökonomischen Anreizes zum Mining. Da die Bitcoin-Preise weitgehend konstant blieben, fiel entsprechend die Hashrate ähnlich wie zuvor deutlich ab.

Überraschenderweise stieg jedoch bereits eine Woche später die Hashrate erneut deutlich an, ohne dass die Preise für Bitcoin in änlichem Umfang stiegen. Dies könnte folgende Ursachen haben:

- Bei einer genaueren Betrachtung der erwarteten Gewinne durch Mining müssen auch Transaktionsgebühren, die der Erzeuger eines neuen Blocks bekommt, einbezogen werden – insbesondere nach dem Halving machten diese an manchen Tagen bis zu 20 % der Belohnung aus und die Transaktionsgebühren sind nach dem Halving auch teilweise deutlich angestiegen.
- Die Difficulty wird nicht in Echtzeit, sondern nur etwa alle 14 Tage angepasst – mit der Difficulty vor dem Halving lohnte es sich also ggf. nicht, am Mining teilzunehmen, dafür aber mit der dann erstmalig nach dem Halving angepassten und damit deutlich verringerten Difficulty (es dauert also eine Weile, bis sich das System wieder im Gleichgewicht befindet).
- In China beginnt im Mai und Juni in einigen Regionen die Regenzeit, sodass durch Wasserkraft viel billiger Strom verfügbar ist und einige Mining-Pools Strom bereits für knapp 0,03 USD/kWh anbieten, insbesondere da sich der Wettbewerb angesichts der sinkenden Mining-Erlöse deutlich verschärft hat [26].
- Es wird verstärkt moderne, energieeffiziente Hardware erworben und eingesetzt, wodurch die variablen Kosten signifikant sinken.

**Figure 2:**
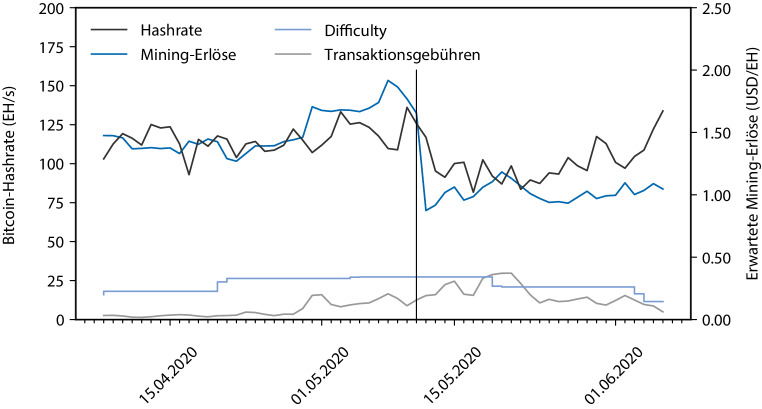

Um diese Zusammenhänge zu untersuchen, analysieren wir den Zusammenhang zwischen dem ökonomischen Anreiz, am Mining teilzunehmen, in Form eines erwarteten Mining-Erlöses in USD je $10^{\mathsf{18}}$ berechneter Hashes und der tatsächlichen Partizipation in Form der Hashrate, im Zeitraum um das Halving mit einem genaueren Modell. Dieses berücksichtigt auch die tatsächliche momentane Blockzeit bzw. Difficulty sowie die zusätzlich zu den Block Rewards vom Erzeuger eines neuen Blocks eingenommenen Transaktionsgebühren. Die sich daraus ergebenden Verläufe sind in Abb. 2 dargestellt. Dabei zeigt sich, dass die Korrelation zwischen erwarteten Gewinnen und der Hashrate mit ca. 0,57 sehr hoch ist. Dies legt nahe, dass bereits viele Miner in Echtzeit oder kurzfristig entscheiden, ob sich für sie aktuell die Teilnahme am Mining lohnt oder nicht, weil sie bereits vor dem Halving nahe an der Kostenneutralität operiert haben. Neben den Unregelmäßigkeiten um das Halving sind vor allem in den letzten 2 dargestellten Wochen geringere Korrelationen zu verzeichnen, dies könnte durch die beschriebenen geänderten Stromtarife in Chinas Mining-Pools bedingt sein.

**Figure 3:**
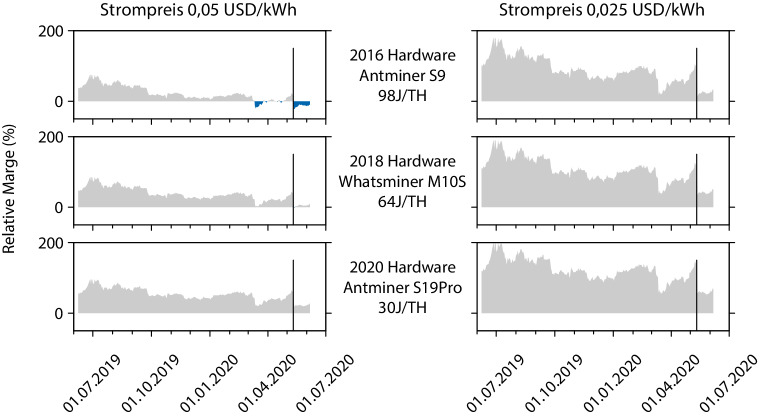

Um den Einfluss der Stromtarife noch näher zu analysieren, betrachten wir in Abb. 3 den Einfluss von Mining-Hardware und Strompreisen auf die relative Marge, also das Verhältnis von Mining-Gewinnen (d. h. die Differenz aus Mining-Erlösen und Stromkosten) zu den Stromkosten des Mining im Detail. Dabei wird ausschließlich der Stromverbrauch berücksichtigt; weitere variable Kosten sowie Investitionen (bspw. für Hardwarebeschaffung) werden ignoriert. Dadurch entsprechen die gezeigten Daten einer Obergrenze der relativen Marge. Als Referenzhardware wurde für Hardware, die im Jahr 2016 neu auf den Markt kam, der weitverbreitete Bitmain Antminer S9 (11,5 TH), für 2018 der MicroBT Whatsminer M10S und für 2020 der Bitmain Antminer S19 Pro (110 TH) herangezogen [27]. Diese entsprachen zum Zeitpunkt ihrer Markteinführung wohl jeweils der energieeffizientesten Hardware. Der senkrechte Strich zeigt wie in Abb. 1 und Abb. 2 den Zeitpunkt des Bitcoin Halving. In der Tat wird bei Strompreisen von 0,05 USD/kWh durch das Halving alte, weniger energieeffiziente Hardware kurzfristig aus dem Markt gedrängt, wohingegen modernere, energieeffizientere Hardware profitabel bleibt und bei niedrigeren Strompreisen auch das Mining mit älterer Hardware ökonomisch sinnvoll bleibt.

**Figure 4:**
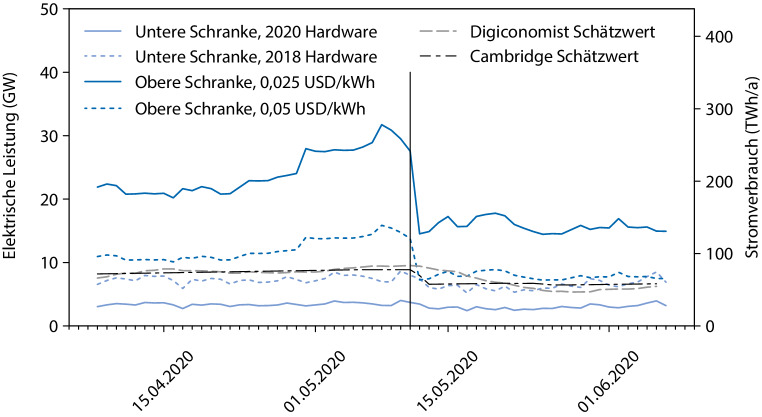

Aufgrund der Beobachtungen ist naheliegend, dass die hergeleitete obere Schranke mittlerweile wohl tatsächlich ein guter Schätzer für den tatsächlichen Stromverbrauch von Bitcoin ist. Abb. 4 zeigt die sich aus den unterschiedlichen Szenarien ergebenden Stromverbräuche von Bitcoin. Die untere Schranke mit 2020 Hardware sowie die obere Schranke mit 0,025 USD/kWh sind als sehr verlässlich anzusehen, da kaum davon auszugehen ist, dass nennenswerte Mining-Aktivitäten mit effizienterer als der modernsten auf dem Markt erhältlichen Hardware oder Stromkosten unter 0,025 USD/kWh stattfinden. Realistischer sind aber auf Basis tatsächlich im Netzwerk typischer Hardware und Strompreise die beiden gestrichelten Schranken. Die Schätzwerte von Digiconomist [28] und Cambridge [29] scheinen angesichts der sicheren oberen und unteren Schranken zwar plausibel und reihen sich gut in diese realistischen Schranken ein, erklären aber möglicherweise den Einbruch der Hashrate infolge des Kurssturzes und des Bitcoin Halving nicht hinreichend. Insofern könnte man durchaus erwarten, dass sich die tatsächliche Hashrate vor dem Halving eher an der oberen Schranke für 0,05 USD/kWh orientiert hat und nach dem Halving aufgrund des verschärften Wettbewerbs durch billige Stromtarife auch Mining-Hardware, die zunächst aus dem Markt gedrängt wurde, wieder eingesetzt wurde und damit der tatsächliche Stromverbrauch sogar über die obere Schranke mit 0,05 USD/kWh gestiegen ist.

Man könnte jedoch erwarten, dass dies nur ein vorübergehender Effekt ist und sich der Stromverbrauch nach dem Ende der Regenzeit eher wieder an der oberen Schranke mit 0,05 USD/kWh orientiert. Selbst wenn sich der Stromverbrauch von Bitcoin aufgrund sehr niedriger Strompreise im Bereich von weniger als 0,025 USD/kWh aufhalten sollte, so ist davon auszugehen, dass es sich in diesem Fall um Strom aus erneuerbaren Energien handelt, da deren Grenzkosten auch 0 sein können, wohingegen die Kosten für Stromerzeugung durch fossile oder nukleare Brennstoffe kaum 0,025 USD/kWh unterschreiten dürften. Insofern würde selbst eine Überschreitung der „sicheren“ oberen Schranke aufgrund lokal oder vorübergehend niedriger Strompreise wohl keinen höheren CO_2_-Fußabdruck für Bitcoin bedeuten als mit der oberen Schranke mit 0,025 USD/kWh.

Unter der Annahme gleichbleibender ökonomischer Rahmenbedingungen, also der Preise für Bitcoin und Strom sowie der Transaktionsgebühren, bedeutet diese Orientierung des tatsächlichen Stromverbrauchs an der oberen Schranke auch, dass durch die periodischen Halvings der Stromverbrauch von Bitcoin langfristig deutlich sinken wird. Aktuell machen die Block-Rewards noch etwa 80 % der Mining-Erlöse aus, bei gleichbleibenden Preisen für Strom und Bitcoin sowie Transaktionsgebühren würde demnach in 4, 8 bzw. 12 Jahren der Stromverbrauch im Vergleich zu Anfang 2020 um 40 %, 60 % und 70 % zurückgehen und sich langfristig bei etwa 20 % des heutigen Wertes, also 20 TWh/a und damit ca. 4 % des heutigen deutschen Stromverbrauchs, einpendeln. Da ökonomische Rahmenbedingungen wie die Preise für Bitcoin und Transaktionsgebühren praktisch nicht verlässlich vorhergesagt werden können, stellt dies keine verlässliche Prognose dar. Jedoch kann man daraus schließen, dass bereits in wenigen Jahrzehnten und bei beschränkten Bitcoin-Preisen der Wert der durch Bitcoin verbrauchten elektrischen Energie nicht mehr wesentlich größer sein wird als die kumulativen Transaktionsgebühren. Etwa ab dem Jahr 2140 wird das ohnehin der Fall sein; ob Bitcoin so lange relevant sein wird, ist jedoch nach nur 11 Jahren kaum zu beurteilen. Durch die Konkurrenz zu anderen Kryptowährungen sowie technische Weiterentwicklungen, etwa hinsichtlich der Performanz (Verringerung der Knappheit von Transaktionen), könnte man vermuten, dass sich auch die Transaktionsgebühren im Vergleich zum heutigen Niveau nicht mehr nennenswert erhöhen werden. Diese Argumentation kann man auf viele Kryptowährungen, die wie Bitcoin eine periodische Halbierung der Block-Rewards aufweisen, übertragen.

## Alternative Konsensmechanismen

Allgemein setzt sich der Stromverbrauch von Blockchains aus 2 Komponenten zusammen: dem Konsensmechanismus, also dem Prozess, in dem sich die Nodes einigen, welche Transaktionen in welcher Reihenfolge ausgeführt werden, und dem redundanten Ausführen der Transaktionen an sich, also etwa dem Verifizieren von Signaturen und dem Anpassen von „Kontoständen“ in der lokalen Datenbank jedes Node (State Transitions). Bei Bitcoin sorgt der PoW-Konsensmechanismus wie beschrieben für den enormen Stromverbrauch; der kumulative Aufwand für das redundante Ausführen der Transaktionen ist im Verhältnis dazu selbst bei der aktuellen Größe des Netzwerks von ca. 10.000 Nodes vernachlässigbar [3]. Seit der Entwicklung von Bitcoin wurden jedoch – unter anderem wegen des hohen Stromverbrauchs von PoW – alternative Konsensmechanismen entwickelt. Für nicht zugangsbeschränkte Systeme ist die bislang wohl erfolgreichste Alternative der Proof-of-Stake-(PoS-)Konsensmechanismus. Hier wird das Stimmgewicht nicht an erbrachte Rechenleistung, sondern an die ebenfalls knappe und innerhalb des Netzwerks sichtbare und damit überprüfbare Ressource Kapital, also besessene Einheiten der Kryptowährung gekoppelt. Oft muss für die Teilnahme an der Abstimmung Kapital für einen gewissen Zeitraum „eingefroren“ werden und dient dann als Sicherheit, um einen ökonomischen Anreiz für korrektes Verhalten der Nodes zu schaffen. Bei PoS entfällt somit der Wettbewerb um immer mehr Rechenleistung. Der mit dem Konsensmechanismus verbundene Energiebedarf ist entsprechend im Vergleich zu PoW vernachlässigbar und erfordert nur wenige Operationen auf jedem Node. Beispiele für aktuelle Implementierungen von Blockchains mit PoS-Konsensmechanismus sind Algorand, Cardano, EOS und Tezos. Daneben wird für Ethereum, die nach Marktkapitalisierung zweitgrößte Kryptowährung, wohl noch im Laufe dieses Jahres eine umfassende Weiterentwicklung, Ethereum 2.0 (Serenity) in Betrieb gehen, die ebenfalls auf PoS basiert und langfristig die aktuelle Ethereum-Blockchain ablösen bzw. integrieren soll [30]. Neben rein PoS-basierten Blockchains gibt es auch Kombinationen von PoS und wahlbasierten Protokollen, wie beispielsweise der für das Cosmos-Netzwerk verwendete Konsensmechanismus Tendermint. Auch in solchen Protokollen ist kein besonders rechen- bzw. energieintensiver Prozess enthalten.

Es gibt unter Anhängern von PoW und PoS häufig Diskussionen, ob PoS genauso sicher ist wie PoW – es gibt dabei gute Argumente für beide Seiten. Beispielsweise sind langfristig bei PoW aufgrund von Economies of Scale beim Mining (Beschaffung von Hardware und elektrischer Energie) und standortabhängiger ökonomischer Rahmenbedingungen Zentralisierungseffekte zu erwarten, was die aktuell starke Zentralisierung von Bitcoin-Mining bestätigt. Dies wiederum kann Sicherheitsprobleme nach sich ziehen. Bei PoS entspricht dagegen die Teilnahme am Konsensmechanismus einer Verzinsung des eingesetzten Kapitals, sodass das Verhältnis des Kapitals aller Teilnehmer am Konsensmechanismus und damit auch das Gewicht ihrer Stimme konstant bleiben. Auf der anderen Seite kann man bei PoW auch am Konsens teilnehmen, ohne wie bei PoS zunächst an Ressourcen aus dem Netzwerk selbst kommen zu müssen, was bei einem bereits stark zentralisierten System das Redezentralisieren deutlich erschweren könnte. Zudem ermöglichen viele PoS-Systeme aktuell nur ab einer gewissen Mindesteinlage die eigene Teilnahme am Konsensmechanismus und für PoW-Blockchains existiert auch eine einfachere Entscheidungsregel, welche Blockchain im Falle eines Konflikts (Forks) die „gültige“ ist. Der Erfolg von PoS-Blockchains in den letzten Jahren sowie Forschung auf diesem Gebiet [31] deuten darauf hin, dass PoS eine vergleichbar hohe Sicherheit wie PoW gewährleisten kann.

Im Kontext von Konsortien im privaten oder öffentlichen Sektor verwendete, zugangsbeschränkte Blockchains verwenden allesamt wahlbasierte Konsensmechanismen, die teilweise als crash-fault-tolerante Vereinfachungen (etwa RAFT) oder auch byzantine-fault-tolerante (etwa PBFT oder RBFT) Nachfolger von Paxos angesehen werden können. Auch deren konsensbezogener Energieverbrauch ist damit wie bei PoS vernachlässigbar klein.

## Redundante Operationen

Unabhängig von der Art der Konsensmechanismen sind alle Blockchains durch redundante Datenhaltung und Operationen charakterisiert. Entsprechend sind der damit verbundene kumulative Rechenaufwand und damit Stromverbrauch – wenn man von hardwareseitigen Unterschieden absieht – proportional zu der Teilnehmerzahl im Blockchain-Netzwerk. Für kleine Blockchain-Netzwerke, wie sie typischerweise im zugangsbeschränkten Kontext in Konsortien verwendet werden, ergibt sich durch die Redundanz im Vergleich zu einem zentralen System ein vielfacher Stromverbrauch. Dies muss jedoch nicht bedeuten, dass der Einsatz einer Blockchain aus Nachhaltigkeitsperspektive negativ sein muss. Folgende grobe Abschätzung soll das veranschaulichen: Ein kleines, privates Blockchain-Netzwerk, wie etwa Hyperledger Fabric oder Quorum, mit 10 Nodes und jeweils mittelmäßiger Hardwarekonfiguration (2 CPUs, 8 GB RAM) kann problemlos 1000 einfache Transaktionen pro Sekunde abwickeln. Dies bedeutet je Transaktion einen Energieaufwand in der Größenordnung von höchstens 1 J. Auf der anderen Seite lässt sich aus den im Nachhaltigkeitsbericht 2017 von VISA genannten Angaben berechnen, dass der Energieverbrauch des gesamten Unternehmens (also inklusive des Heizens von Gebäuden etc.) heruntergerechnet ca. 6000 J je Transaktion beträgt, von denen für Datencenter ca. 3000 J je Transaktion anfallen [32]. Ein simples Client-Server-System mit einfachem Key-Value-Store, etwa LevelDB, kann mit der oben genannten Hardwareausstattung mehrere Tausend simple Transaktionen pro Sekunde abwickeln, was zu einem Stromverbrauch in der Größenordnung von 0,02 J pro Transaktion führt. Ensprechend ist zwar der Stromverbrauch einer Blockchain aufgrund der Redundanz (und zu einem Teil auch aufgrund des Konsenses sowie allgemein der umfänglicheren Verwendung von kryptografischen Methoden) in der Regel deutlich höher als der einer ensprechenden zentralen Lösung (hier um den Faktor 50), macht aber möglicherweise dennoch nur einen sehr kleinen Teil des Stromverbrauchs der gesamten IT-Lösung bzw. des kompletten Prozesses aus, selbst wenn man noch Clients und Back-ups mit einbezieht. Insbesondere in Szenarien, in denen mithilfe von energieeffizienten Varianten der Blockchain-Technologie Prozesse weiter digitalisiert werden können, ist es also nicht abwegig, dass auch blockchain-basierte Lösungen unter dem Strich Energie einsparen können.

In den bekanntesten Kryptowährungen, wie etwa Bitcoin und Ethereum, bestehen die entsprechenden Blockchain-Netzwerke bereits jetzt aus vielen Tausenden Nodes und deren Anzahl dürfte bei großflächiger Adoption in Zukunft stark steigen. Entsprechend kann man den Stromverbrauch dieser Netzwerke wegen des resultierenden hohen Grades an Redundanz durchaus als problematisch betrachten. Auch für diese Herausforderung hält jedoch die Forschung vielversprechende Lösungskonzepte bereit: Grundsätzlich wird durch reduzierten Grad an Redundanz, also das Nachrechnen von Transaktionen nur auf Teilmengen aller Nodes, auch der Stromverbrauch pro Transaktion gesenkt. Dies ist etwa bei sogenannten Second-Layer-Konzepten wie Lightening oder Raiden der Fall, jedoch typischerweise mit Tradeoffs bei der Sicherheit verbunden, da diese ja gerade mit auf dem hohen Grad an Redundanz beruht. Ähnlich wird es bei der bereits angesprochenen Ethereum‑2.0‑Blockchain eine Aufteilung des Netzwerks in insgesamt 64 sogenannte Shards geben, die durch eine Hauptkette, die sogenannte Beacon Chain, periodisch referenziert werden und dadurch bei jeder solchen Referenzierung auch die Sicherheit des Gesamtsystems erben. Bis Ethereum 2.0 diese Features vollumfänglich nutzt und entsprechend Prozesse auf die verschiedenen Shards verteilt sind, wird noch einige Zeit vergehen, daher ist es zum gegenwärtigen Zeitpunkt schwer zu quantifizieren, wie stark der Grad an Redundanz letztlich sinkt und welchen Einfluss dies auf Sicherheit und Funktionalität haben wird.

## ZK-Rollups

Besonders vielversprechend sind aktuell Fortschritte im Zusammenhang mit in den vergangenen Jahren – vor allem durch Blockchain-Technologie in den Fokus gerückten – „Proofs of Computational Integrity“, die man im Blockchain-Umfeld vielleicht besser unter dem Schlagwort „Zero Knowledge Proofs“ (ZKP) kennt. Damit ist es möglich, (probabilistisch) mit einem in der Regel sehr kurzen und leicht zu verifizierenden Beweis zu zeigen, dass bestimmte Berechnungen korrekt ausgeführt wurden, ohne alle Details der Rechnung angeben bzw. die komplette Berechnung wiederholen zu müssen. Zunächst wurden ZKP von manchen Kryptowährungen wie bspw. Zcash eingesetzt, um die bei Kryptowährungen wie Bitcoin praktisch nicht vorhandene Vertraulichkeit von Transaktionen wiederherzustellen [33].

Eine wesentliche Eigenschaft von typischen ZKP ist, dass die Größe von Beweisen und die rechnerische Komplexität der Verifizierung der Beweise in der Regel sublinear (bspw. konstant oder polylogarithmisch bei SNARKS bzw. STARKS [34, 35]) mit der Größe der zu verifizierenden Berechnung skalieren. Dadurch ist es möglich, dass eine einzelne Partei, beispielsweise eine Kryptobörse, in sogenannten ZK-Rollups eine Vielzahl (mehrere Tausend oder Zehntausend von Transaktionen) bündelt und nur einen kurzen Beweis, dass alle Schritte korrekt ausgeführt wurden (Prüfen von Signaturen, korrektes Aktualisieren von Kontoständen …), als Transaktion an die Blockchain schickt, wo der Beweis von den übrigen Knoten mit geringem Aufwand geprüft wird. Im Detail können sich die Architekturen hier deutlich unterscheiden, sowohl hinsichtlich der genutzten ZKP-Technologie als auch der weiteren auf der Blockchain gespeicherten und kontinuierlich zu aktualisierenden Daten. (Hier gibt es einen Tradeoff: Weniger Daten auf der Blockchain bedeutet eine höhere Abhängigkeit von der Partei, die die Beweise erstellt und die Kontostände aller Accounts bei sich aktualisiert und speichert, aber auch höhere Skalierbarkeit, da die Blockchain dann keinen Engpass darstellt.)

Im Gegensatz zu bislang bestehenden Second-Layer-Lösungen können ZK-Rollups mit kompletter On-Chain-Datenhaltung dieselben Sicherheitsgarantien wie die Blockchain selbst gewährleisten, da der Beweis nach wie vor von allen Teilnehmern am Netzwerk geprüft wird und so Manipulationen mit derselben Sicherheit wie herkömmliche Transaktionen ausgeschlossen werden können [36]. Zudem können erhebliche Verbesserungen im Vergleich zu der herkömmlichen Verarbeitung von Transaktionen erreicht werden, da der Großteil der Speicher- und Rechenkapazität auf digitale Signaturen zurückgeht, deren Überprüfung der Betreiber des ZK-Rollups in einen kurzen Beweis komprimiert. In bestehenden Prototypen werden mithilfe von ZK-Rollups bereits Transaktionsraten von mehreren Hundert bis mehreren Tausend Transaktionen pro Sekunde in Ethereum (herkömmlich ca. 10 Transaktionen pro Sekunde) erreicht [37], [38]. Wir wollen hier exemplarisch abschätzen, welche Implikationen dies auf den durch Redundanz bedingten Anteil des Stromverbrauchs eines großen Blockchain-Netzwerks haben kann.

Auf Basis des sich bereits im Einsatz befindenden ZK-Rollups von Loopring auf der Ethereum-Blockchain kann man die Einsparmöglichkeiten im Idealfall, also bei maximaler Auslastung der ZK-Rollups und ausschließlichem Vorhandensein von Transaktionen innerhalb des ZK-Rollups, gut abschätzen: Für Loopring 3 werden die sogenannten Gaskosten, die ein Maß für den Speicher- und Rechenaufwand einer Transaktion in Ethereum sind und die Bepreisung des Ausführens von Transaktionen bestimmen, bei maximaler Auslastung von 2100 Transaktionen pro Sekunde auf 365 beziffert [37]. Zum Vergleich benötigt eine einfache Transaktion in Ethereum mindestens 21.000 Gas, oft auch deutlich mehr. Entsprechend bedeutet dies bereits eine Reduktion der redundanten Operationen und damit deren Strombedarfs pro Transaktion um einen Faktor von ca. 100 (eine exakte Proportionalität von Gaskosten und Rechenaufwand ist nicht gegeben, näherungsweise ist dies aber eine sinnvolle Annahme). Auf der anderen Seite werden die Kosten für die rechenaufwendige, aber nur beim Betreiber des Rollups erforderliche Beweiserstellung mit 0,000042 USD je Transaktion beziffert [37]. Bei 2100 Transaktionen je Sekunde entspricht dies einem Betrag von ca. 5 USD pro Minute und damit beispielsweise dem Betrieb von AWS-Instanzen mit ca. 96 vCPUs. Für diese kann man einen Stromverbrauch von einigen Hundert Watt abschätzen [39, 40], was damit nicht mehr als 0,5 J je Transaktion ergibt. Dies entspricht auch der Größenordnung aus einer alternativen Abschätzung, die zumindest für ZKP-freundliche Hashfunktionen einen um den Faktor 100 erhöhten Aufwand für die Beweiserstellung im Vergleich zu einer einfachen Berechnung angibt. Auf der anderen Seite ergeben eigene Messungen mit ethereum-basierten Blockchains ohne PoW-Konsensmechanismus einen Stromverbrauch von ca. 0,01 J je Transaktion (tx) und Node aus der CPU-Nutzung. In einem Netzwerk von ca. 10.000 Nodes, was in etwa der heutigen Größe des Bitcoin- und Ethereum-Netzwerks entspricht, ergibt sich damit ohne ZK-Rollup ein Stromverbrauch von ca. 100 J/tx. Mit ZK-Rollup dagegen erhält man für die redundanten Operationen, also in erster Linie das Verifizieren des Beweises, einen Betrag von ($100/100+0{,}5$) J/tx und damit eine Energieersparnis von 98,5 %. Für noch größere Netzwerke würde sich die Energieeinsparung in unserem Beispiel entsprechend auf bis zu 99 % vergrößern, wohingegen sie für kleinere Netzwerke sinkt und für Netzwerke mit nur sehr wenigen Nodes sogar zu einer Erhöhung des Stromverbrauchs führen würde.

Jedoch muss in einer ganzheitlichen Betrachtung auch der Leerlaufstrom von Nodes berücksichtigt werden. Je nachdem, ob extra für die Teilnahme an der Blockchain ein eigener PC verwendet wird bzw. angeschaltet bleibt oder der PC ohnehin läuft bzw. in einer Cloud mit tendenziell niedrigem Leerlaufstrom angesiedelt ist, sowie abhängig von der durchschnittlichen Auslastung des Blockchain-Netzwerks kann der Leerlaufstrom für große Netzwerke in Nicht-PoW-Blockchains die im Zusammenhang mit Transaktionen aufgewendeten Energiemengen deutlich übersteigen oder aber dagegen vernachlässigbar sein. Weitere Verbesserungen bei der Energieproportionalität könnten dazu führen, dass Leerlaufstrom in Zukunft weniger Bedeutung haben wird [41]; große Datencenter sind hier in der Regel bereits weiter fortgeschritten als kleine [42]. Zudem ist ein Szenario, in dem alle Transaktionen innerhalb eines ZK-Rollups abgewickelt werden, unrealistisch. Dennoch ist denkbar, dass ein Großteil von Finanztransaktionen innerhalb solcher ZK-Rollups abgewickelt werden kann. ZK-Rollups wurden in erster Linie entwickelt, um Skalierbarkeits- und Performanceprobleme von Blockchains zu lösen, und haben wie eben beschrieben den angenehmen Nebeneffekt, dass sie auch zu erheblichen Verbesserungen beim Stromverbrauch beitragen können. Diese sind jedoch nur dann spürbar, wenn kein PoW-basierter Konsensmechanismus bereits einen so hohen Stromverbrauch verursacht, dass sich Verbesserungen bei den redundanten Operationen überhaupt nicht bemerkbar machen, und der Leerlaufstrom absolut oder im Verhältnis zu dem durch Transaktionen verursachten Stromverbrauch aus CPU-Nutzung vernachlässigbar ist.

## Fazit

Die aktuell zahlreichen Kryptowährungen zugrunde liegenden PoW-Blockchains und insbesondere Bitcoin haben – unter Berücksichtigung ihrer derzeitigen technischen Performanz – einen enormen Energieverbrauch. Der gesamte Stromverbrauch all dieser PoW-Kryptowährungen wird nach wie vor zum größten Teil durch Bitcoin verursacht und beträgt zwischen 20–50 % des deutschen Stromverbrauchs, mit einem Best Guess für Bitcoin bei ca. 100 TWh/a bzw. 20 % des deutschen Stromverbrauchs. Dabei ist die treibende Kraft hinter dem Stromverbrauch der Preis von Bitcoin und nicht etwa die Anzahl an Transaktionen und bei gleichbleibenden ökonomischen Rahmenbedingungen würde langfristig durch die bei vielen PoW-basierten Kryptowährungen auftretenden periodischen Halbierungen der Block-Rewards auch der entsprechende Stromverbrauch deutlich zurückgehen.

Zudem gibt es mittlerweile etablierte Blockchains mit alternativen Konsensmechanismen, vor allem PoS für offene, nicht zugangsbeschränkte Kryptowährungen und die wahlbasierten Konsensmechanismen privater, zugangsbeschränkter Blockchains. Unter Letztere fallen in der Regel Lösungen, die etwa in Unternehmen oder dem öffentlichem Sektor als organisationsübergreifende, neutrale Plattform eingesetzt werden. Aufgrund des Wegfalls des PoW ist deren Stromverbrauch jeweils um Größenordnungen geringer als der von Bitcoin und anderen PoW-basierten Kryptowährungen. Vor allem aufgrund der für Blockchain-Technologie charakteristischen, redundanten Berechnungen ist jedoch deren Stromverbrauch pro Transaktion in etwa proportional zur Anzahl teilnehmender Knoten und damit immer noch um ein Vielfaches höher als der von zentralen Systemen. Insbesondere für große Kryptowährungsnetzwerke kann dies auch für Nicht-PoW-Blockchains noch immer einen hohen Stromverbrauch bedeuten. Durch technologische Weiterentwicklungen und Modifikationen, mit denen der Aufwand für redundante Berechnungen und Datenhaltung gesenkt werden kann, und insbesondere Zero-Knowledge-Proofs in ZK-Rollups, ist zu erwarten, dass in Zukunft der Stromverbrauch großer Netzwerke noch einmal deutlich gesenkt werden kann. Bei einer ganzeitlichen Betrachtung muss jedoch auch stets der Leerlaufstrom berücksichtigt werden.

**Figure 5:**
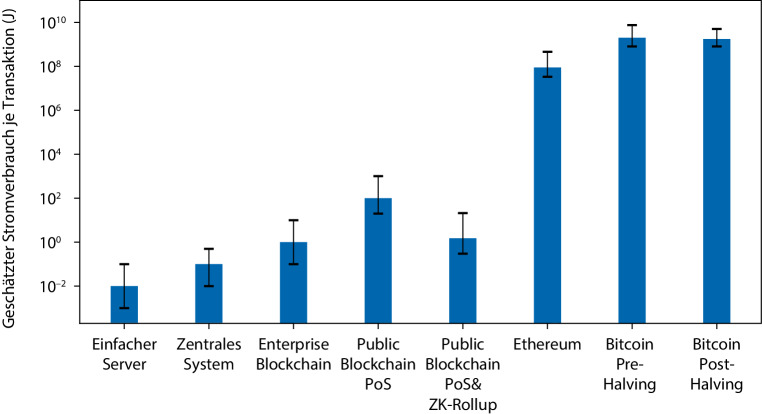

Abb. 5 fasst die Erkenntnisse dieses Beitrags durch eine Abschätzung typischer Stromverbräuche von unterschiedlichen, in den vorherigen Abschnitten beschriebenen Blockchain-Technologien bzw. Verbesserungen zusammen. Diese dienen in erster Linie der Veranschaulichung und berücksichtigen nicht den Leerlaufstrom. Die angegebenen Zahlenwerte sollten daher noch nicht als feststehend und verlässlich betrachtet werden, sondern nur als größenordnungsmäßige Anhaltspunkte. Insbesondere die Fehlerabschätzungen sind in den meisten Fällen nicht allgemein belastbar, da sie Erfahrungswerten aus Tests mit verschiedenen Systemen entsprechen. Die Größenordnungen sind jedoch auf Basis der in den jeweiligen Kapiteln gemachten Annahmen nach bestem Wissen der Autoren sinnvolle Abschätzungen. Abb. 5 illustriert, dass der Stromverbrauch von bereits heute in der Anwendung genutzten Blockchains verglichen mit den PoW-Blockchains der „ersten Generation“ um mehrere Größenordnungen verringert ist bzw. mit heute verfügbarer Technologie sein könnte.

Auch wenn die Verwendung von Blockchain-Technologie aus rein technischer Sicht nicht die energieeffizienteste Lösung sein mag, kommt es letztlich darauf an, welche Energieeinsparungen im Gegenzug durch den Einsatz der Technologie erzielt werden können. Das Potenzial im branchen- und wirtschaftszweigübergreifenden Einsatz von IT ist dabei besonders groß [43]. Gerade hier setzen auch viele der über Kryptowährungen hinausgehenden Blockchain-Anwendungen, die aktuell in Wirtschaft und öffentlichem Sektor entwickelt oder erprobt werden, an. Denn aus ökonomischen oder politischen Gründen ist in diesen Szenarien oft keine zentrale, digitale Plattform durchsetzbar. Bei der Verwendung der beschriebenen, energieeffizienten Blockchain-Lösungen ist davon auszugehen, dass bei der damit verbundenen Automatisierung von Prozessen nicht nur aus finanzieller, sondern auch aus energetischer Sicht Ressourcen eingespart werden können.

Hier ist es Aufgabe der Wirtschaftsinformatik, die vorhandenen Potenziale für Energieeinsparungen und Klimaschutz zu erkennen, zu quantifizieren und – je nach Szenario mit oder ohne Blockchain-Technologie – mit der geeignetsten Technologie zu heben. Voraussetzung hierfür ist, dass der Verbrauch von Ressourcen (bspw. durch eine CO_2_-Steuer) so bepreist wird, dass keine ökologisch schädlichen Verzerrungen auftreten und deshalb die ökonomischen Anreizmechanismen eine Entwicklung solcher Lösungen fördern. Zudem sollte man sich auch stets darüber im Klaren sein, dass eine zunächst aufgrund der hohen Komplexität und der noch jungen Technologie teuer erscheinende Lösung, die insgesamt den Ressourcenverbrauch durch den Einsatz moderner Lösungen wie Blockchain reduziert, auch eine Absicherung gegen zukünftige Preiserhöhungen oder -schwankungen sein kann [44].

Abschließend wollen wir daher zu weiterer Forschung sowohl im Bereich technischer Verbesserungen von Blockchain-Technologie, etwa hinsichtlich Performanz und Energieeffizienz, als auch zu Einsatzgebieten mit besonders hohem Potenzial für Energieeinsparungen ermutigen.
